# Heart rate agreement between the 20-meter shuttle run test and virtual system in healthy children: a cross-sectional study

**DOI:** 10.1186/s12887-019-1861-4

**Published:** 2019-12-12

**Authors:** Cristiane A. Moran, Simone Dal Corso, Maria Teresa Bombig, Andrey Jorge Serra, Silvana Alves Pereira, Maria Stella Peccin

**Affiliations:** 1 Departamento de Ciências da Saúde, Federal University of Santa Catarina, Campus Jardim das Avenidas, Rod. Gov. Jorge Lacerda, 3201, Araranguá, Santa Catarina CEP 88.906-072 Brazil; 2 Nove de Julho University, São Paulo, Brazil; 3 Federal University of São Paulo, São Paulo, SP Brazil; 4 Federal University of Rio Grande do Norte, Natal, Rio Grande do Norte Brazil

**Keywords:** Cardiology, Child, Exercise, Physical fitness, Virtual reality, Incremental stress test

## Abstract

**Background:**

Earlier studies evaluated the physiological responses to video games in children with different clinical conditions; however, no study has compared active video games with an incremental field test in healthy children. The purpose of this study was to verify the agreement between the 20-m shuttle run test (20 m-SRT) and virtual system (VS).

**Methods:**

This is a cross-sectional study of 235 children (9.0 ± 0.8 years, 109 boys). The two tests were performed one week apart and the children were instructed not to engage in any physical exercise or sports in the 24 h preceding each test. Their resting heart rate was monitored for one minute and then throughout the tests. To evaluate the influence of motivation on the 20 m SRT and (VS), at the end of the tests the children were asked to rate their motivation on a scale of zero to 10, zero being “not cool” and 10 “awesome”. Perceived exertion at the end of the tests was assessed using the modified Borg scale.

**Results:**

Maximum heart rate (HRmax) did not differ between the 20 m-SRT and VS (194.4 ± 10.2 bpm vs. 193.2 ± 13.8 bpm, respectively). Both tests were similar for intensity > and < 96% HRmax. The children showed greater exertion on the Borg scale and motivation during the VS. The multiple logistic regression model showed that motivation (*p* = 0.98), sex (*p* = 0.53), age (*p* = 0.61), nutritional status (*p* = 0.65), and speed (*p* = 0.18) were not predictive factors of the child’s reaching HRmax.

**Conclusion:**

VS can be used as a tool to evaluate the intensity of maximal exercise tests, given that the percentage of children who achieved HRmax did not differ between the VS and 20 m SRT. The perceived exertion scales were correlated, but only the modified Borg scale correlated with HRmax in the 20 m SRT. The tests are motivational, and most children obtained the maximum VS score.

## Background

In recent years, interactive video games, known as Virtual System (VS) [1], have been used for rehabilitation in different areas, such as geriatrics [2], neurology [3–5] and pediatrics [6–8].

Earlier studies have evaluated the physiological responses to video games in healthy children or those with pulmonary disease [8, 9]. In pediatrics, due to the motivational aspect [10, 11], researchers have used virtual systems as a resource to assess cardiac response. However, the findings of a recent systematic review demonstrate that the children in these studies did not reach maximum exercise intensity [12].

It is known that immediately before and at the onset of exercise, cardiovascular changes induced by the neural command center located in the bulbar region occur, resulting in increased heart rate and myocardial contractility [13].

Heart rate (HR), which represents intensity during exercise [14], is considered maximum above 180 bpm in children, but the maximum threshold used is 200 bpm [15, 16], while Pfeiffer considers a variation between 185 and 225 bpm [17].

The latest systematic reviews [12, 18] of studies on children playing interactive video games have shown an association between an increase in exercise intensity from mild to moderate and a rise in HR. However, it is still unknown whether VS promotes vigorous intensity during a field test, such as the 20-m shuttle run test (20 m-SRT).

The field test most widely used to assess aerobic capacity in healthy schoolchildren is called the 20 m-SRT. Created in 1982 by Luc Léger et al., it was modified and validated in 1984 and is currently recommended for children and adolescents by several specialists in the area [19–22].

However, in the health area, researchers started to develop new interventions that use active video games [18, 23], indicating them in exercise tests as a health indicator in children and to assess cardiac capacity, using the 20 m-SRT [24, 25].

The present study was based on the hypothesis that VS would offer a new approach to incremental testing in order to assess maximum heart rate (HRmax), considering possible equivalence between the tests. The aim of this study was to verify the agreement between 20 m-SRT and VS.

## Methods

### Study design and sample

State elementary schools in the city of Sao Paulo, Brazil were randomly contacted to select healthy students aged 8 to 10 years, for this analytical cross-sectional study.

The ethics committee of the Federal University of São Paulo approved the study (protocol no. 1860/10. The parents gave written consent to take part in the research.

The sample size was calculated based on results obtained in an earlier pilot study conducted with six children that used HRmax as outcome variable. Thus, a sample of 235 children was obtained under the assumption that in the test comparing pairs of means (Student’s t-test) for dependent samples, the standard deviation “σ” for HRmax would be 21.16 bpm, with a difference in HRmax between the SRT and VS of 6.33 bpm. We considered a power of 90% and an error of 0.05%.

### Protocol

The city of Sao Paulo, Brazil is divided into five regions: east, west, north, south and center. We randomly contacted 5 schools, one from each of these regions, two of which declined to participate. A total of 312 children from the three remaining institutions were invited, 294 of whom completed the questionnaires on medical history and physical activity. Seventeen children were excluded for failing to attend on the day of medical screening, one child refused to be examined, and 34 required further investigations for exhibiting clinical and resting 12-lead electrocardiogram (ECG) changes. These children were referred to the outpatient clinic for congenital heart defects of the Federal University of Sao Paulo. Of the 242 eligible children, seven who did not appear for testing were considered losses and the remaining 235 were included in the final sample.

The parents completed a questionnaire on the child’s clinical condition and any family history of heart disease. Next, a cardiologist assessed each child, recording their medical history and conducting a physical examination. Blood pressure was measured and a resting ECG was obtained, in order to rule out cardiovascular disease or clinical changes that could hinder physical activity.

### Evaluations

Anthropometric data were used to characterize the sample and the variables were measured according to the Brazilian Pediatric Society’s Orientation Manual (2009) [26].

### Body mass index

Body mass index (BMI) was expressed as Z-score [27, 28] (Table 1). Body weight was measured with the child barefoot, wearing pants and a shirt. Digital scales (G-life®, Magna, China) were used, with a maximum load of 150 kg and resolution of 100 g. Height was measured using a wall-mounted measuring tape, with the children barefoot, feet parallel and together, standing upright with their arms extended to the side and head positioned such that the lower part of the eye socket was at the same level as the earhole [26].

**Table 1:** Sample description

Variable		N	(%)/ Mean/SD	*p* value	
Gender	Female	126	53.6	0.26	
	Male	109	46.4		
Age (years)			9.0/±0.8	0.01	
Anthropometry	Weight (kg)		35.8/± 9.9	0.01	
	Height (m)		1.4/± 0.1	0.20	
zBMI	Low weight (z score < −2)	0		0.50	
	Normal (−2 ≤ z score < + 1)	195	83		
	Overweight (+ 1 ≤ z score < + 2)	28	12		
	Obese (z score > + 2)	12	5		

% = percentage; kg = kilograms; m = meters; *N* = sample; *SD* = standard deviation. zBMI: Body mass index (BMI) expressed as Z score

### Shuttle run test

In the 20-m shuttle run test, participants run 20 m. The test starts at a standard speed of 8.0 km/h, increasing 0.5 km/h every minute. Participants were advised of the test pace and a beeping sound signaled progression to the next level. The step rate was maintained by a standardized recorded beep played on a Toshiba TR8172MU CD player placed 10 m from the subject. The children were instructed to complete as many stages as possible. The test was ended when the child did not reach the expected distance, in line with the beep, and corresponding to the stage executed. The 20 m-SRT was conducted on a flat surface at the multi-sport courts of the schools.

The 20-m course was marked with two cones and the children performed the tests individually to avoid their competing with other subjects. The raters accompanied all the tests as a safety measure and to encourage the children to complete the course within the time limits established for each stage. The children were verbally encouraged during each stage using standard phrases such as “you’re doing really well” and “keep going” in a clear loud voice to guarantee total comprehension. The completed stages were converted into meters [21, 29].

### Virtual system

The VS test was conducted in the school video rooms using the Nintendo Wii® Free Run video game (Nintendo Company Ltd., Kyoto, Japan, model RVLSWC/RVLSWFSP), part of the Wii Fit Plus set of games. Free Run consists of producing a virtual running field. The Free Run game takes place on an island in the presence of virtual participants, known as avatars. The children run the race at a steady self-determined pace for 20 min, with no obstacles or change in intensity.

A predefined path sets the distance in the game, and since an avatar that represents the player in the virtual environment provides the route, the distance in the real world has no relation to the one presented in the game. The player’s movements are shown on three accelerometers in the Wii motion plus® control. The static race provides body movements in the horizontal and vertical plane and the higher the exercise intensity, the greater the distance covered. To ensure reliable results, the researchers monitored the children throughout the test according to the manufacturer’s instructions, guiding the placement of the controller near the body.

Test run time was determined in the VS for a maximum of twenty minutes, pre-established by the investigator for the free racing game. Distance was converted from miles to meters and speed was calculated based on time and distance.

Similarly to the 20 m-SRT, the raters accompanied the tests to ensure the children completed the course until the end of the game and to provide verbal encouragement.

To eliminate any possible motivational influence from the VS, the order of the tests was randomized and the child was asked to choose an envelope, to determine which test (VS or 20 m-SRT) would be performed first. The envelopes were opaque, sealed and numbered sequentially. The children performed the tests one week apart and were instructed not to engage in any physical exercise or sports in the 24 h preceding each test.

### Heart rate

The children were monitored at rest for one minute and then throughout the tests. The variables used for statistical analysis were resting HR and HRmax. A Polar RS800CX® heart rate monitor was set up using Polar ProTrainer 5® software to collect 1-s samples of heartbeats and R-R intervals, in line with the manufacturer’s recommendations for maximum accuracy.

The HRmax data used were the values on the heart monitor display at the end of the exercise. Data stored on the heart monitor were transferred to the software at the end of the data collection sessions and the memory cleared to make room for further data collection.

Data recorded on Polar ProTrainer 5® software served only for visual verification of the R-R curve. The chart indicated that heart rate increased during the tests and remained around HRmax at the end of the exercise. This initial check was used to ensure that a 1-s sample time was not affected by noise coming from the device.

Resting HR was assessed while the children were sitting in a comfortable position with their back and lower limbs supported for 60 s, after a 5-min rest. HRmax, defined as the maximum value attained during the 20 m-SRT and VS tests, was 200 bpm [30]. HRmax was also expressed as percentage of the maximum predicted using the equation $\left(\frac{HRmax}{200}\right)\ast 100$, which showed a mean HR of 83 and 72% of predicted HRmax in the VS and 20 m-SRT tests, respectively. The change in basal heart rate was also calculated, considering resting heart rate - HRmax achieved in the VS, expressed as a percentage:

$$ {}^{\varDelta}\mathrm{maximum}\ \mathrm{HR}\ \mathrm{achieved}\to \mathrm{resting}\ \mathrm{HR}=\frac{\left(\mathrm{maximum}\;\mathrm{HR}\;\mathrm{achieved}-\mathrm{resting}\;\mathrm{HR}\right)\;}{\mathrm{resting}\;\mathrm{HR}}\ast 100 $$

Continuous heart rate monitoring was used to analyze maximum heart rate during the 20 m-SRT and VS tests.

### Motivation

To evaluate the influence of motivation on the execution of the 20 m-SRT and VS, the children were asked to rate their motivation at the end of the tests, on a scale of zero to 10, zero being “not cool” and 10 “awesome”. Perceived exertion at the end of the tests was assessed using the modified Borg scale.

## Statistical analysis

The Kolmogorov-Smirnov test was applied to determine how well heart rate (resting and maximum), the scale of perceived exertion, and motivation adhered to normal distribution. Descriptive statistics were expressed as means and standard deviations for the numerical variables, and as absolute and relative frequencies for their categorical counterparts. The chi-squared test was used to evaluate the intensity achieved in both tests. The paired Student’s t and Wilcoxon tests were applied to compare the 20 m-SRT and VS for cardiac output and motivational factors, respectively. The independent Student’s t-test was applied to compare the perceived exertion obtained with the 20 m-SRT and VS. Multiple logistic regression was performed to analyze cardiac output by observing whether the child had reached HRmax (≥ 200 bpm), using sex, age, nutritional status, motivation and speed in the VS as predictors. Bland-Altman analysis was applied to examine the agreement between the two methods that measure the same parameter. It evaluated the difference in means (BIAS) between 20 m-SRT and VS, to show the agreement between them. The Bland-Altman plot contains three lines: the center line being the difference in means, and the upper and lower lines the limits of agreement, which are calculated as ±1.96 x SD of the difference in means between both methods. The influence of starting the tests with the shuttle run or the VS was analyzed using the nonpaired Student’s t-test. A 95% confidence interval and significance level of *p* < 0.05 were used for all analyses. Statistical information was obtained with SPSS statistical software, version 20.0 (SPSS, Chicago, IL, USA).

## Results

A total of 235 children were included in the study, 118 and 117 of whom started on the VS and 20 m-SRT, respectively. There was no difference in HRmax response (*p* = 0.056) between the order in which the tests were performed. The sample is characterized in Table 1.

In addition, we observed that both tests were similar for intensity > and < 96% of predicted HRmax (*p* = 0.50), Table 2.

**Table 2:** Intensity > 96% of maximum HR reached on the tests

		SV	
		Yes	No
SRT-20 m	Yes	118	43
	No	36	38

SRT-20 m = shuttle run teste de 20 metros; *SV* = virtual system; % = percentagem

**Fig. 1:**
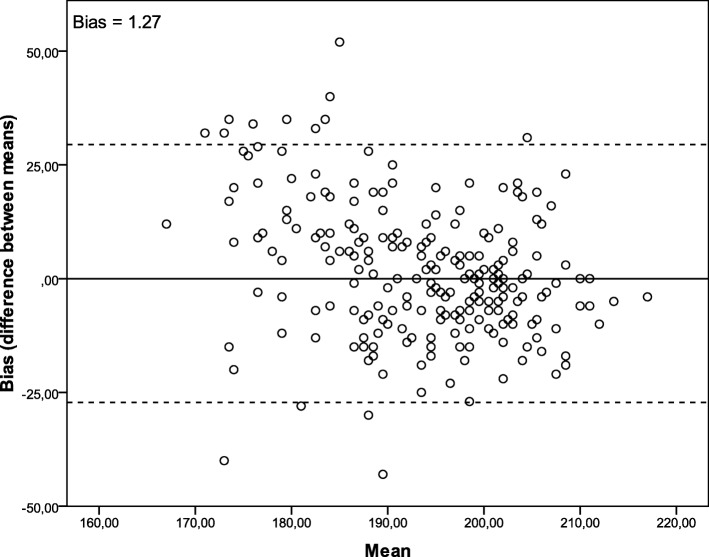
Methodological agreement by Bland-Altman analysis for HRmax between VS and 20m-SRT. The y-axis (Bias) is the difference between heart rate means and the x-axis (Mean) is the individual mean for two tests. The bias was 0.1 log (*p* = 0.18)

The children’s performance between the 20 m-SRT and VS tests showed no difference in HRmax (*p* = 0.18). The difference between the means is illustrated on a Bland-Altman plot (Fig. 1).

A comparison of the children’s performance between the tests showed that there was no difference in HRmax (*p* = 0.18). To assess HRmax agreement between VS and 20 m-SRT, we plotted the differences against the mean for each subject using a Bland-Altman analysis. The mean difference was 0.1 (*p* = 0.18) (Fig. 1).

According to the results obtained, the number of children motivated in both tests was statistically significant (*p* < 0.001). However, most of the children (84%) obtained the maximum score [10] in the VS.

All children reached HRmax in 10 ± 5 min. Exertion was greater on the Borg scale in the VS (7 points) compared to the 20 m-SRT (6 points) (*p* = 0.002), and 30 (12.8%) children were unable to complete the VS due to signs of exhaustion.

The multiple logistic regression model showed that motivation (*p* = 0.98), sex (*p* = 0.53), age (*p* = 0.61), nutritional status (*p* = 0.65), and speed (*p* = 0.18) were not predictive factors of the child’s reaching HRmax ≥200 bpm.

The order of starting the tests, whether the 20 m-SRT (117 children) or VS (118 children), showed no differences in maximum (*p* = 0.056) and mean HR (*p* = 0.208).

## Discussion

A similar percentage of children reached HRmax in both tests. There was no difference in HRmax between tests, but mean HR was higher in the VS. The tests were similar in terms of intensity > and < 96% of predicted HRmax. With respect to Borg scale scores, there was greater exertion in the VS when compared to the 20 m-SRT, but the children were motivated in both tests.

The characteristics chosen for the VS, such as free running with no obstacles or change in intensity, allowed the children to dictate the pace of the exercise. This approach provided a level of intensity corresponding to 83% of estimated HRmax, a value similar to that recommended by the American College of Sports Medicine for prescribing training for children [9, 31].

High-intensity, constant-load exercise protocols are commonly used to assess children’s tolerance to exercise [32]. Despite not being an incremental protocol, our findings demonstrate that the VS is an innovative exercise test, with a constant load profile, where heart response reaches maximum intensity. The children reached maximum HR after 10 min, a time similar to that found in protocols using incremental tests, such as the Bruce protocol adapted for children [33, 34].

Physical effort acts directly on HR and is linearly related to exercise intensity [35, 36], exhibiting a strong correlation with maximal oxygen uptake (VO_2_ max). As described in an earlier study, VO_2_ max is reached when maximum HR reaches 180 bpm [37]. Although we did not take precise measures of maximum oxygen intake, the children may have reached VO_2_ max, since the maximum heart rate was above 200 bpm.

The increase in heart rate during exercise can be explained by the rise in sympathetic and decline in parasympathetic tone in healthy children [38, 39]. This expected response was observed in the VS, since we found a 120% increase from resting to maximum heart rate during the test, which was higher than that reported in studies with games that depend primarily on the lower limbs, whose values ranged from 51 to 98% [12].

Mean heart rate also contributes to a better understanding of the cardiovascular system’s response. Straker found a mean HR of 130 bpm in a video game, a value similar to that obtained in basketball and soccer [40]. In his study, children achieved a mean HR of 166 bpm in the VS. Using this line of reasoning, we can prescribe video games as a physical activity.

Health promotion aims at a healthy lifestyle via aerobic training starting in childhood [41], and the basic concept when prescribing physical activity to children is to encourage regular exercise, prioritizing an entertaining environment [42].

The characteristics of the game selected for the VS, that is, free running without obstacles or with changes in intensity, is one of the strong points of the study, since the children themselves determined the pace of the exercise, thereby enabling 83% of estimated maximum HR, a value above that recommended by the American College of Sports Medicine for prescribing physical activity in children [9, 31].

Studies recommend video games with an appropriate frequency and duration for physical training [10, 43, 44]. Thus, the device seems to contribute to physical activity, resulting in an appropriate exercise intensity for children [44], which contradicts Graves [31], demonstrating that video games were not enough to improve their cardiorespiratory fitness [31].

In adults, the perceived exertion scales show an association with signs of exhaustion and physiological measurements that include the musculoskeletal and cardiopulmonary systems. According to some researchers, applying perceived exertion scales in small children is difficult, because the rate of cognitive maturation depends on the age of the child and due to the fact that numerical interpretation of perceived exertion is not easily achieved [45–47].

The feeling of tiredness is considered a subjective response that can be influenced by several factors such as the clarity of instructions and the cognitive ability of the individual [48]. According to Prasad [49], the use of subjective assessments in children requires them to know the meaning of “out of breath” [49]. Homerding [48], believes that the modified Borg scale is adequate from the age of 9 years and suggests that the greater the child’s functional impairment, the greater the respiratory discomfort and the easier the understanding of perceived exertion [48].

Children younger than 9 years old have difficulty interpreting perceived exertion scales [48, 50], which may be due to their phase of cognitive development. These findings were confirmed in the present study, since only maximum HR was correlated with the modified Borg scale in the 20 m-SRT.

Studies demonstrate that the VS is a motivational instrument for physical activity and that children are motivated by a number of factors during a video game. These include the challenge of finishing the game, reaching objectives and their interest in the game, in addition to the sensory experience and their control over events [51–53].

Although there are no validated scales to assess children’s motivation, the subjects of this study were demonstrably motivated by the tests. As such, new technologies, such as the interactive games of the VS, provide a motivational stimulus to children [54], a fact observed here, since most of the players scored higher in the VS compared to the shuttle run test.

The main study limitation was the children’s difficulty in interpreting the perceived exertion scale, which can be explained by the age range selected. Although two schools refused to participate, restricting the sample to only three regions in the city of São Paulo, we obtained a representative sample.

The findings demonstrate that the VS can be implemented as an alternative assessment instrument for maximum exercise intensity for children in school settings and outpatient facilities, without the need for large spaces or substantial investment in equipment. Moreover, video games contribute to the motivational aspect of children because it is an entertaining and easy-to-understand instrument that can be used in rehabilitation programs, possibly leading to greater adherence of children to physical activities.

For future research, the authors suggest that studies be designed as clinical trials to rehabilitate children with cardiorespiratory and metabolic disorders in addition to the area of pediatric oncology. We also recommend studying physiological responses with an emphasis on the upper and lower limbs, according to the style of the game selected.

## Conclusions

The VS can be used as a tool to evaluate the intensity of maximal exercise tests given that the percentage of children who achieved HRmax did not differ between the instrument and the 20 m-SRT. The perceived exertion scales were correlated, but only the modified Borg scale correlated with HRmax in the 20 m-SRT. The tests are motivational, and most children obtained the maximum score in the VS.

## Data Availability

The dataset used and analyzed during the current study is available from the corresponding author on reasonable request.

## Abbreviations

20 m- SRT: 20-m shuttle run test
BMI: Body mass index
bpm: Beats per minute
HRmax: Maximum heart rate
VO_2_max: Maximal oxygen uptake
VS: Virtual system
